# Supplementary material for: RhoGEF2 overexpression induces cell competition dependent on Ptp10D, Crumbs and the Hippo signaling pathway
Source: J Cell Sci. 2025 Oct 14;138(19):jcs264377. doi: 10.1242/jcs.264377 (PMC12579956; doi:10.1242/jcs.264377)
Supplement: Supplementary information [file joces-138-264377-s1.pdf]

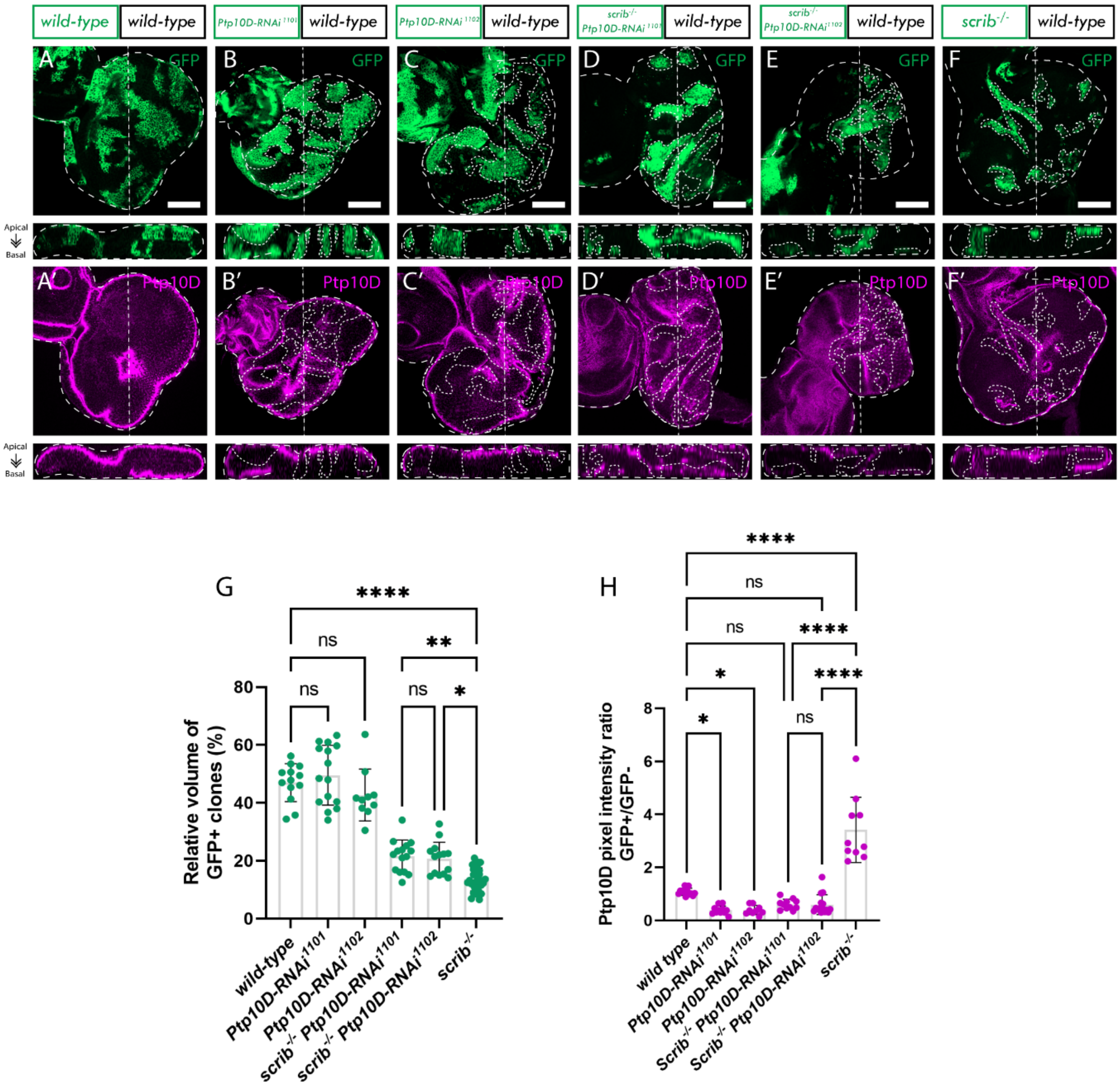

Fig. S1. Ptp10D is required for *scrib*$^{-/-}$ cell elimination. A-F, eye discs *of ey-FLP-MARCM*-induced mosaics (clones are marked by the presence of GFP and *wild-type* tissue is unmarked): **A)** *wild-type*; **B)** *UAS-Ptp10D-RNAi* (VDRC 1101 line); **C)** *UAS-Ptp10D-RNAi* (VDRC 1102 line); **D)** *scrib*$^{-/-}$ *UAS-Ptp10D-RNAi* (VDRC 1101 line); **E)** *scrib*$^{-/-}$ *UAS-Ptp10D-RNAi* (VDRC 1102 line)*;* **F)** *scrib*$^{-/-}$. **A′-F′)** Ptp10D immunostains from A-F. **G)** Quantifications of relative GFP+ clone volume [*wild-type* (n=13); *Ptp10D-RNAi*^{1101} (n= 15); *Ptp10D-RNAi*^{1102} (n=10); *scrib*$^{-/-}$ *Ptp10D-RNAi*^{1101} (n=15); *scrib*$^{-/-}$ *Ptp10D-RNAi*^{1102} (n=14); *scrib*$^{-/-}$ (n=32); Statistical test used were one-way ANOVA, p<0.05, with Tukey's multiple comparison test. In graph: p*: 0.0143; **: 0.0026; ****: p<0.0001]. **H)** Quantification of Ptp10D immunostain pixel intensity ratio between GFP+ and GFP- clones [*wild-type* (n=13); *Ptp10D-RNAi* ^{1101} (n= 11); *Ptp10D-RNAi*^{1102} (n=9*); scrib*$^{-/-}$ *Ptp10D-RNAi*^{1101} (n=11); *scrib*$^{-/-}$ *Ptp10D-RNAi*^{1102} (n=15); *scrib*$^{-/-}$ (n=10); Statistical test used were one-way ANOVA, p<0.05, with Tukey's multiple comparison test. In graph: *: 0.0188 (*wild-type* vs *Ptp10D-RNAi*^{1101}); *: 0.0325 (*wild-type* vs *Ptp10D-RNAi*^{1102}); ****: p<0.0001]. ns = not significant; error bars = SD; scale bars indicate 50 µm. Images below show an *xz* cross section of the corresponding eye-antennal disc from the apical (top) to basal (bottom) edge, with the position of the chosen *xz* sections indicated by a vertical dotted line in the *xy* images. Note that folding at the edges of the discs can result in Ptp10D being observed basally in the *xz* sections in these regions, despite the staining still being localized at the apical membrane. Dotted lines surrounding discs or clones illustrate disc/clone boundaries. Note that Fig. S1A, D, E, F = Fig. 1A-D, and are shown here to highlight the clones for the Ptp10D immunostaining.

Journal of Cell Science • Supplementary information

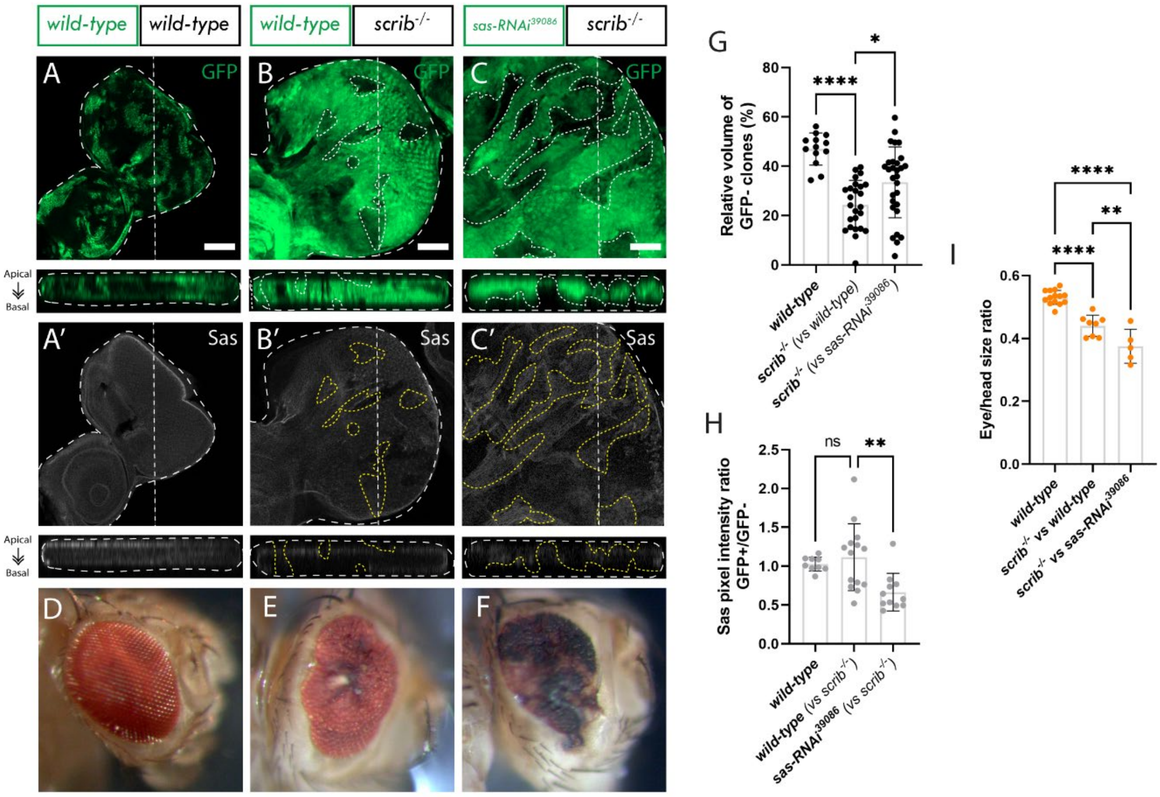

Fig. S2. Sas, in *wild-type* neighbouring cells, is necessary for the elimination of *scrib$^{-/-}$* clones in eye disc mosaics. A-C, eye-antennal discs of *ey-FLP-reverse-MARCM*-induced mosaics (clones are marked by the absence of GFP and wild-type tissue marked with GFP) bearing: **B)** *scrib$^{-/-}$* (GFP-) and *wild-type* (GFP+); **C)** *scrib$^{-/-}$* (GFP-) and *Sas-RNAi^{39086}* (GFP+). Note the *reverse MARCM* stock contains *FRT82B scrib$^{-/-}$ Tub-GAL80* and therefore the standard *MARCM* stock was used as the control. The control disc may be smaller relative to the *scrib$^{-/-}$* mosaic discs due to *scrib$^{-/-}$* clones inducing non-cell autonomous proliferation of the *wild-type* tissue. **A'- C')** Sas immunostains from A - C. **D – F)** Adult eye mosaics corresponding to above panels; **G)** Quantification of relative GFP- volume [*wild-type* vs *wild-type* (n=13); *scrib$^{-/-}$* vs *wild-type* (n=25); *scrib$^{-/-}$* vs *Sas-RNAi^{39086}* (n=29); Statistical test used were one-way ANOVA, with Tukey's multiple comparison test. In graph: *:0.0149; ****<0.0001]; **H)** Quantification of Sas pixel intensity ratio between GFP+ and GFP- clones [*wild-type* vs *wild-type* (n=9); *scrib $^{-/-}$* vs *wild-type* (n=14); *scrib$^{-/-}$* vs *Sas-RNAi^{39086}* (n=11); Statistical test used were Kruskal-walis test, with Dunn's multiple comparison test. In graph: **:0.0058]; **I)** Relative eye to head size quantification [*wild-type* vs *wild-type* (n=14); scrib$^{-/-}$ vs *wild-type* (n=8); scrib$^{-/-}$ vs *Sas-RNAi^{39086}* (n=5); Statistical test used were one-way ANOVA, with Tukey's multiple comparison test. In graph: **:0.0056; ****<0.0001]. ns = not significant; error bars = SD; scale bars indicate 50 µm. Below each image is an xz cross section of the corresponding eye-antennal disc from the apical (top) to basal (bottom) edge, with the position of the chosen xz sections indicated by a vertical dotted line in the xy images. Dotted lines surrounding discs or clones illustrate disc/clone boundaries. Adult eye images were taken at the same magnification.

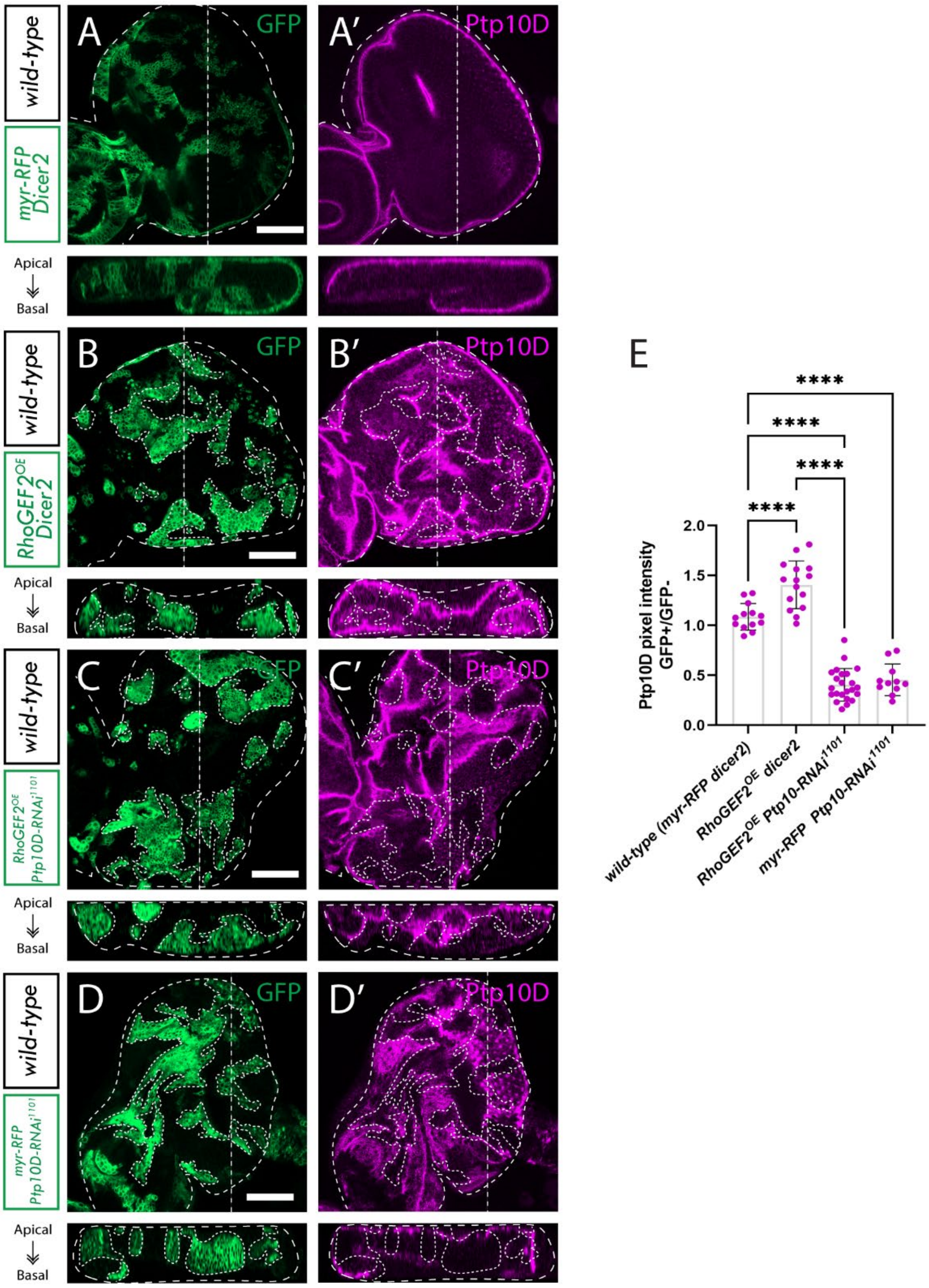

Fig. S3. ***Ptp10D^{KD}* increases *RhoGEF2^{OE}* clonal growth, in *RhoGEF2^{OE}* eye disc mosaics. A-D**, eye discs of *ey-FLP-MARCM*-induced mosaics (clones are marked by the presence of GFP and *wild-type* tissue is unmarked): **A)** *wild-type;* **B)** *UAS-RhoGEF2 UAS-Dicer-2;* **C)** *UAS-RhoGEF2 UAS-Ptp10D-RNAi* (VDRC 1102 line); **D)** *UAS-myr-RFP UAS-Ptp10D-RNAi* (VDRC 1101 line). **A'-D')** Ptp10D immunostains from A-D. **E)** Quantification of Ptp10D pixel intensity ratio between GFP+ and GFP- clones [*wild-type* (n=13); *RhoGEF2^{OE} Dicer2* (n=15); *RhoGEF2^{OE} Ptp10D-RNAi* 1101 (n=23); *myr-RFP UAS-Ptp10D-RNAi* 1101 (n=11); Statistical test used were one-way ANOVA, p<0.05, with Tukey's multiple comparison test. In graph: ****p <0.0001]. ns = not significant; error bars = SD; scale bars indicate 50 µm. Below each image is an xz cross section of the corresponding eye-antennal disc from the apical (top) to basal (bottom) edge, with the position of the chosen xz sections indicated by a vertical dotted line in the xy images. Note that folding at the edges of the discs can result in Ptp10D being observed basally in the xz sections in these regions, despite the staining still being localized at the apical membrane. Dotted lines surrounding discs or clones illustrate disc/clone boundaries.

Journal of Cell Science • Supplementary information

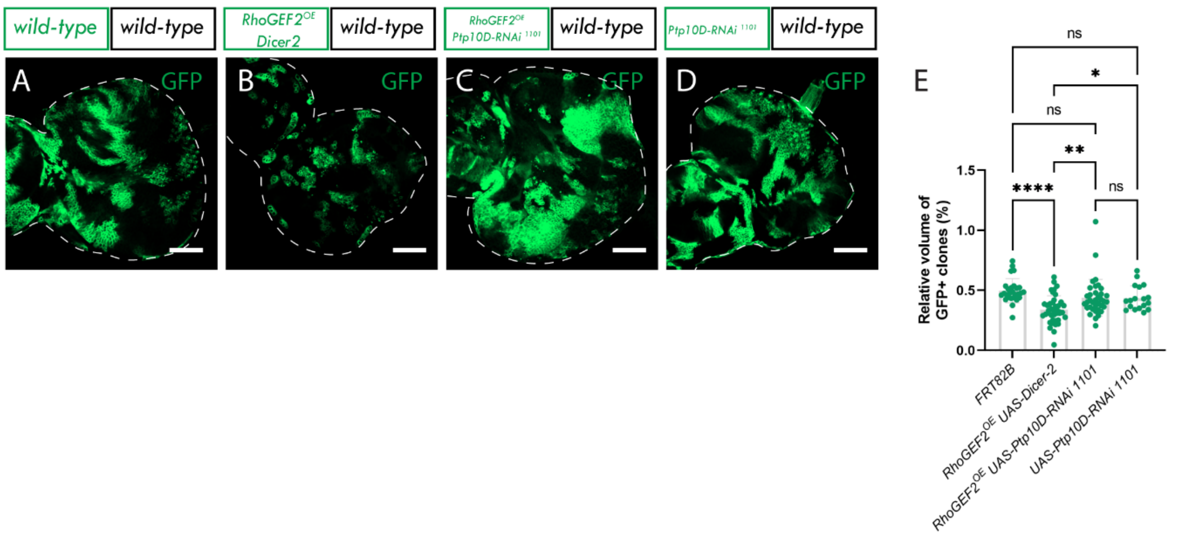

Fig. S4. Moderate overexpressing *RhoGEF2* clones are eliminated by cell-competition in a low-protein diet. A-D, eye discs of *ey-FLP-MARCM*-induced mosaics (clones are marked by the presence of GFP and *wild-type* tissue is unmarked): **A)** *wild-type;* **B)** *UAS-RhoGEF2 UAS-Dicer-2;* **C)** *UAS-RhoGEF2 UAS-Ptp10D-RNAi* (VDRC 1102 line); **D)** *UAS-Ptp10D-RNAi* (VDRC 1101 line). **E)** Quantification of the relative GFP+ clone volume [*wild-type* (n=27); *RhoGEF2^{OE} Dicer2* (n=40); *RhoGEF ^{OE} Ptp10D-RNAi* 1101 (n=35); *myr-RFP UAS-Ptp10D-RNAi* 1101 (n=18); Statistical test used were one-way ANOVA, p<0.05, with Tukey's multiple comparison test. In graph: *: 0.0341; **: 0.0035; ****p <0.0001]. ns = not significant; error bars = SD; scale bars indicate 50 µm. Dotted lines surrounding discs indicate disc boundaries.

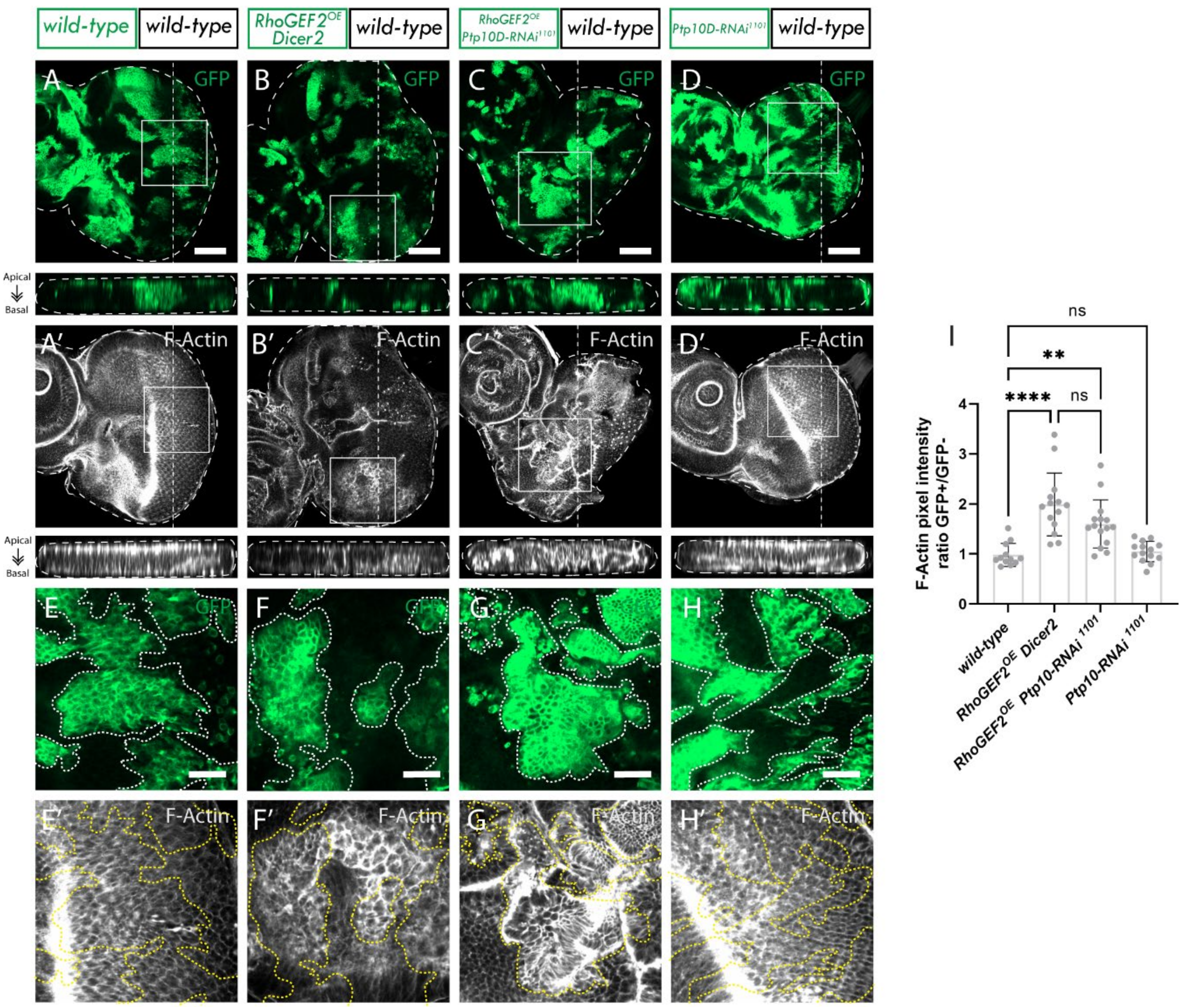

Fig. S5. Knockdown of *Ptp10D* in *RhoGEF2*^{OE} clones does not significantly affect the increased apical F-actin accumulation in *RhoGEF2*^{OE} clones, in eye disc mosaic clones. A-D, eye discs of *ey-FLP-MARCM*-induced mosaics (clones are marked by the presence of GFP and *wild-type* tissue is unmarked): **A)** *wild-type;* **B)** *UAS-RhoGEF2 UAS-Dicer-2;* **C)** *UAS-RhoGEF2 UAS-Ptp10D-RNAi* (VDRC 1102 line); **D)** *UAS-myr-RFP UAS-Ptp10D-RNAi* (VDRC 1101 line); **A'-D')** F-Actin stains from A-D. White squares indicate magnified sections shown below. **E-H)** Magnified sections from A-D. **E'-F')** F-Actin immunostains from E-H; **I)** Quantification of F-Actin pixel intensity ratio between GFP+ and GFP- clones [*wild-type* (n=12); *RhoGEF2*^{OE} *Dicer2* (n=14); *RhoGEF2*^{OE} *Ptp10D-RNAi* 1101 (n=15); *myr-RFP UAS-Ptp10D-RNAi* 1101 (n=14); Statistical test used were one-way ANOVA, $p<0.05$, with Tukey's multiple comparison test. In graph: **0.0063; ****<0.0001]; ns = not significant; error bars = SD; scale bars in A to D indicate 50 µm; scale bars in E to H indicate 20 µm. Dotted lines surrounding discs or clones illustrate disc/clone boundaries

Journal of Cell Science • Supplementary information

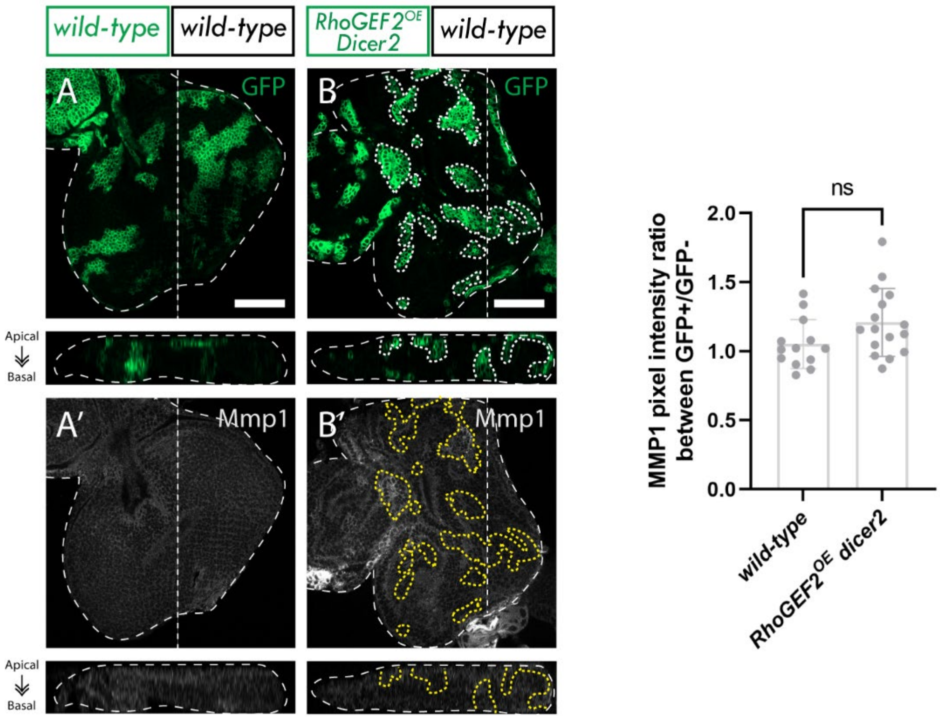

Fig. S6. JNK signaling is not activated by moderate overexpression of *RhoGEF2*. A-B, eye discs of *ey-FLP-MARCM*-induced mosaics (clones are marked by the presence of GFP and *wild-type* tissue is unmarked): **A)** *wild-type;* **B)** *UAS-RhoGEF2 UAS-Dicer-2.* **A'-B')** Mmp1 immunostains from A-B. **C)** Quantification of MMP1 immunostain pixel intensity ratio between GFP+ and GFP- clones [*wild-type* (n=13); *RhoGEF2^{OE} dicer2* (n=16); Statistical test used was unpaired t-test, p<0.05. p=0.0646]. ns = not significant; error bars = SD; Scale bars indicate 50 µm. Below each image is an *xz* cross section of the corresponding eye-antennal disc from the apical (top) to basal (bottom) edge, with the position of the chosen *xz* sections indicated by a vertical dotted line in the *xy* images. Dotted lines surrounding discs or clones illustrate disc/clone boundaries.

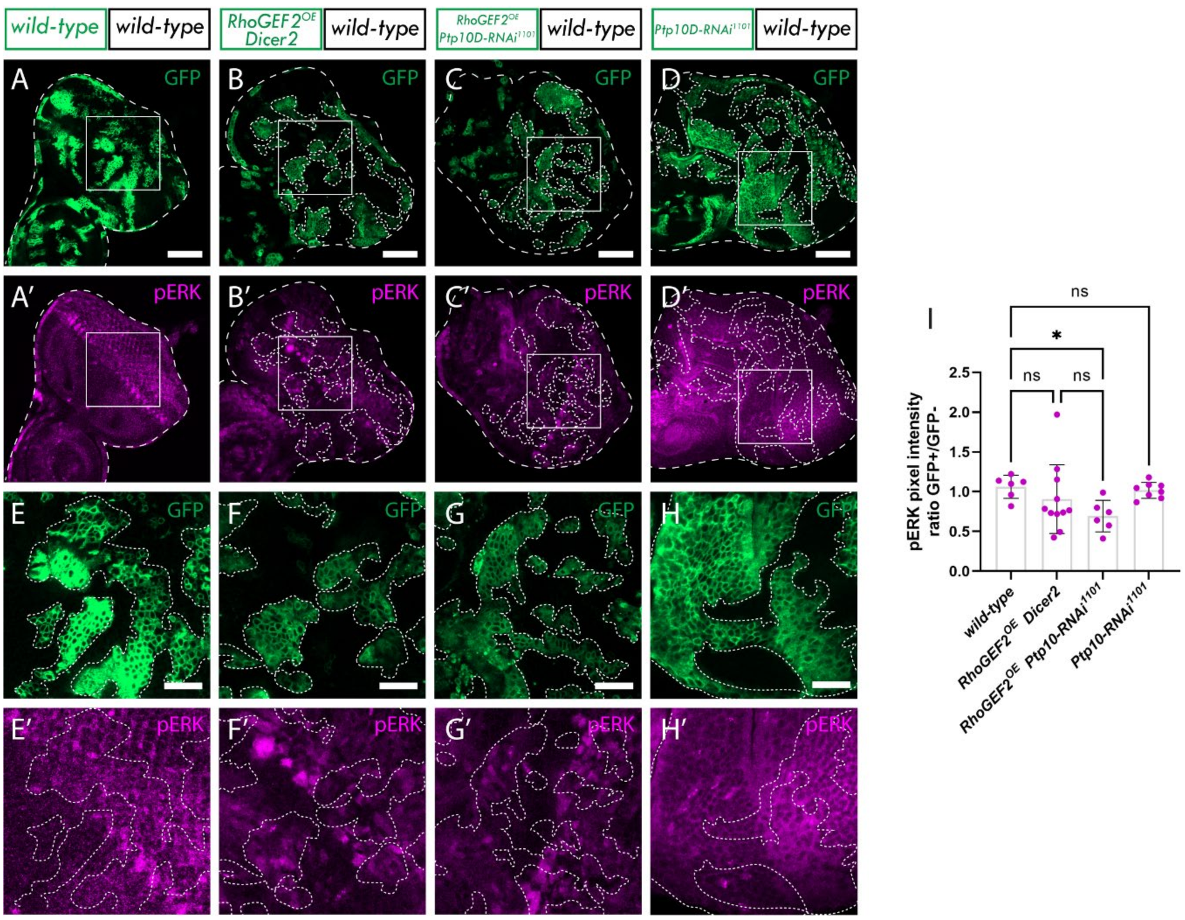

Fig. S7. *Ptp10D* knockdown does not increase Ras signaling in *RhoGEF2^{OE}* clones. **A-D,** eye discs of *ey-FLP-MARCM*-induced mosaics (clones are marked by the presence of GFP and *wild-type* tissue is unmarked): **A)** *wild-type;* **B)** *UAS-RhoGEF2 UAS-Dicer-2;* **C)** *UAS-RhoGEF2 UAS-Ptp10D-RNAi* (VDRC 1102 line); **D)** *UAS-myr-RFP UAS-Ptp10D-RNAi* (VDRC 1101 line); **A'-D')** pERK immunostains from A-D. White squares indicate magnified sections shown below. **E-H)** Magnified sections from A-D. **E'-F')** pERK immunostains from E-F. **I)** Quantification of pERK pixel intensity ratio between GFP+ and GFP- clones [*wild-type* (n=6); *RhoGEF2^{OE} Dicer2* (n=11); *RhoGEF2^{OE} Ptp10D-RNAi* 1101 (n=6); *myr-RFP UAS-Ptp10D-RNAi* 1101 (n=8); Statistical test used were Kruskal-Wallis test, $p<0.05$, with Dunn's multiple comparison test. In graph: *0.0418]; ns = not significant; error bars = SD; scale bars in A to D indicate 50 µm; scale bars in E to H indicate 20 µm. Dotted lines surrounding discs or clones illustrate disc/clone boundaries.

Journal of Cell Science • Supplementary information

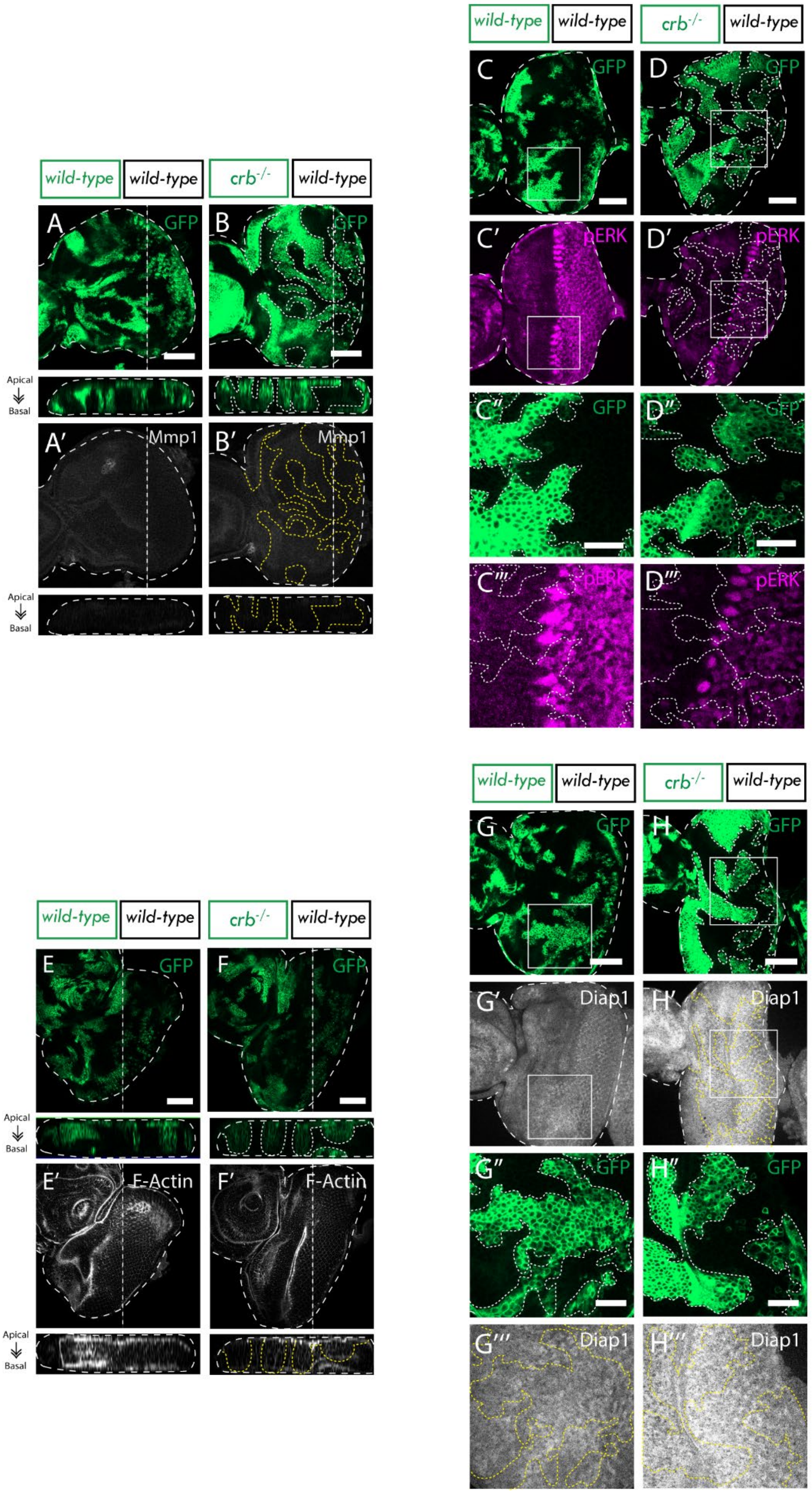
wild-type
wild-type
crb-/-
wild-type
A
GFP
B
GFP
Apical
Basal
A′
Mmp1
B′
Mmp1
C
GFP
D
GFP
C′
pERK
D′
pERK
C″
GFP
D″
GFP
C‴
pERK
D‴
pERK
E
GFP
F
GFP
E′
F-Actin
F′
F-Actin
G
GFP
H
GFP
G′
Diap1
H′
Diap1
G″
GFP
H″
GFP
G‴
Diap1
H‴
Diap1

Fig. S8. Controls for Figure 6. *crb* loss decreases apical F-actin accumulation, rescues the elevated JNK signaling, rescues Ras signaling downregulation and reduces the elevated Hippo signaling in *RhoGEF2^{OE}* clones. A-B, Eye discs of *ey-FLP-MARCM*-induced mosaics (clones are marked by the presence of GFP and *wild-type* tissue is unmarked):
A) *wild-type*; **B)** *crb$^{-/-}$*; **A'-B')** Mmp1 stains from A-B. **C-D,** Eye discs of *ey-FLP-MARCM*-induced mosaics (clones are marked by the presence of GFP and *wild-type* tissue is unmarked): **C)** *wild-type* **D)** *crb$^{-/-}$*; **C'-D')** pERK immunostains from C-D. White squares indicate magnified sections shown in panels below; **C''- D'')** Magnified sections from C-D; **C'''-D''')** pERK imunostains from C''-D''. **E-F,** Eye discs of *ey-FLP-MARCM*-induced mosaics (clones are marked by the presence of GFP and *wild-type* tissue is unmarked): **E)** *wild-type*; **F)** *crb$^{-/-}$*; **E'-F')** F-Actin stains from E-F. **G-H,** Eye discs of *ey-FLP-MARCM*-induced mosaics (clones are marked by the presence of GFP and *wild-type* tissue is unmarked): **G)** *wild-type* **H)** *crb$^{-/-}$*; **G'-H')** pERK immunostains from G-H. White squares indicate magnified sections shown in panels below; **G''- H'')** show magnified sections from G-H; **G''' -H''')** Diap1 stains in G''-H''. ns = not significant; error bars = SD; A, B, C, D, E, F, G, H scale bars indicate 50 µm. C'', D'', G'', H'') scale bars indicate 20 µm. In A, A', B, B', E, E', F and F', images below show an *xz* cross section of the corresponding eye-antennal disc from the apical (top) to basal (bottom) edge, with the position of the chosen *xz* sections indicated by a vertical dotted line in the *xy* images. Dotted lines surrounding discs or clones illustrate disc/clone boundaries.

Journal of Cell Science • Supplementary information

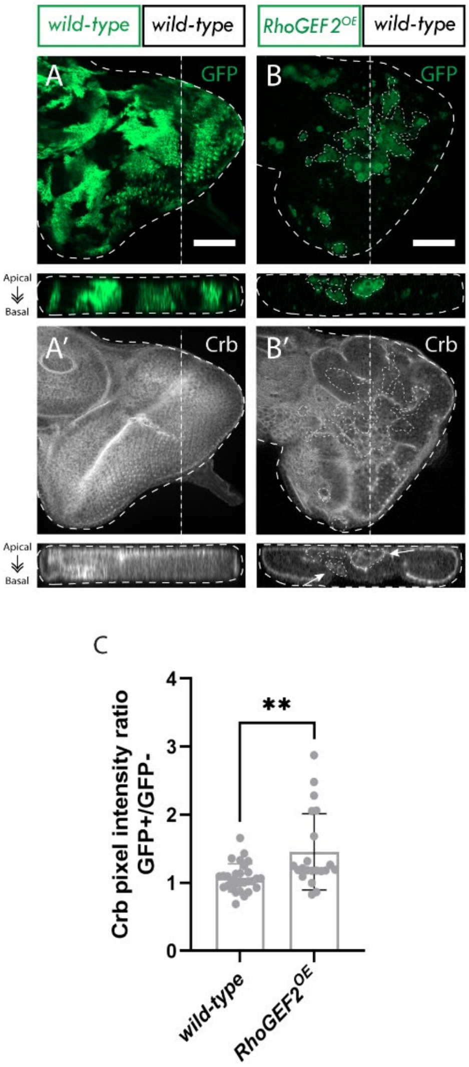

Fig. S9. Crb accumulates in RhoGEF2^{OE} clones. A-B, eye discs of *ey-FLP-MARCM*-induced mosaics (clones are marked by the presence of GFP and *wild-type* tissue is unmarked): **A)** *wild-type*; **B)** *RhoGEF2^{OE}.* **A' - B')** Crb immunostains. Arrows indicate Crb accumulation around GFP+ clones. **C)** Quantification of Crb pixel intensity ratio between GFP+ and GFP- clones [*wild-type* (n=21); *RhoGEF2^{OE}* (n=29); Statistical test used was Mann Whitney Test. In graph: *P-value: 0.0013]. ns = not significant; error bars = SD; scale bars indicate 50 µm. Below each image is an xz cross section of the corresponding eye-antennal disc from the apical (top) to basal (bottom) edge, with the position of the chosen xz sections indicated by a vertical dotted line in the xy images. Note that folding at the edges of the discs can result in Crb being observed basally in the xz sections in these regions, despite the staining still being localized at the apical membrane. Dotted lines surrounding discs or clones illustrate disc/clone boundaries.

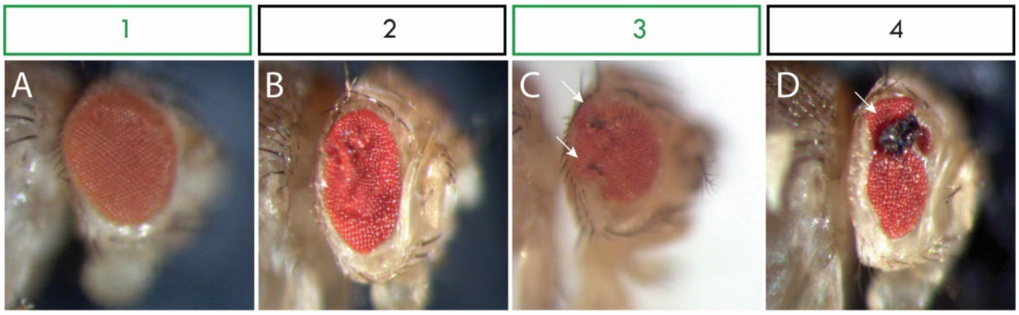

Fig. S10. Representative examples of adult eye severity phenotyping. A) wild-type (severity level 1); B) Slight rough eye (severity level 2); C) Eye with necrotic speckles (arrows indicate necrotic speckles) (severity level 3), D) Rough eye with necrosis (arrow indicates larger necrotic patch) (severity level 4). Note that Fig. S10, panels 2, 3, 4 = Fig. 1G, H, Q, and are shown here to highlight the classification of eye phenotype severity. Adult eye images were taken at the same magnification.

Table S1. Genotypes

Fig 1

A,F,J,O: *eyFLP, UAS-mCD8-GFP /+ ;; tub-GAL4, FRT82B, tub-GAL80/+*

B,G,M,R: *eyFLP, UAS-mCD8-GFP /+ ;; tub-GAL4, FRT82B, tub-GAL80/ FRT82B, scrib*^{1}

C,H: *eyFLP, UAS-mCD8-GFP /+ ;; tub-GAL4, FRT82B, tub-GAL80/ FRT82B, scrib*^{1}*, UAS-Ptp10D-RNAi*^{1101}

D,I: *eyFLP, UAS-mCD8-GFP /+; UAS-Ptp10D-RNAi*^{1102} */ + ; tub-GAL4, FRT82B, tub-GAL80/ FRT82B, scrib*^{1}

K,P: eyFLP, UAS-mCD8-GFP /+; UAS-Ptp10D UAS.cTa / + ; tub-GAL4, FRT82B, tub-GAL80/ FRT82B

L,Q: eyFLP, UAS-mCD8-GFP /+; UAS-Ptp10D UAS.cTa /+ ; tub-GAL4, FRT82B, tub-GAL80/FRT82B, scrib^{1}

Fig 2

A, G: *eyFLP, UAS-mCD8-GFP /+ ;; tub-GAL4, FRT82B, tub-GAL80/+*

B, C, H: *eyFLP, UAS-mCD8-GFP /+ ; UAS-RhoGEF2/ +; tub-GAL4, FRT82B, tub-GAL80/FRT82B*

Fig 3

A, E: *eyFLP, UAS-mCD8-GFP /+ ;; tub-GAL4, FRT82B, tub-GAL80/+*

B, F: *eyFLP, UAS-mCD8-GFP /+ ; UAS-RhoGEF2/+; tub-GAL4, FRT82B, tub-GAL80/UAS- Dicer2*

C, G: *eyFLP, UAS-mCD8-GFP /+ ; UAS-RhoGEF2/+; tub-GAL4, FRT82B, tub-GAL80/UAS-Ptp10D-RNAi*^{1101}

D, H: *eyFLP, UAS-mCD8-GFP /+ ;myr-RFP/+; tub-GAL4, FRT82B, tub-GAL80/UAS-Ptp10D-RNAi*^{1101}

Fig 4

A, E: *eyFLP, UAS-mCD8-GFP /+ ;; tub-GAL4, FRT82B, tub-GAL80/+*

B, F: *eyFLP, UAS-mCD8-GFP /+ ; UAS-RhoGEF2/+; tub-GAL4, FRT82B, tub-GAL80/UAS- Dicer2*

C, G: *eyFLP, UAS-mCD8-GFP /+ ; UAS-RhoGEF2/+; tub-GAL4, FRT82B, tub-GAL80/UAS-Ptp10D-RNAi*^{1101}

D, H: *eyFLP, UAS-mCD8-GFP /+ ;; tub-GAL4, FRT82B, tub-GAL80/UAS-Ptp10D-RNAi*^{1101}

Fig 5

A, E: *eyFLP, UAS-mCD8-GFP /+ ;; tub-GAL4, FRT82B, tub-GAL80/+*

B, F: *eyFLP, UAS-mCD8-GFP /+ ; UAS-RhoGEF2/ +; tub-GAL4, FRT82B, tub-GAL80/FRT82B*

C, G: *eyFLP, UAS-mCD8-GFP /+ ; UAS-RhoGEF2/ +; tub-GAL4, FRT82B, tub-GAL80/FRT82B, crb*^{11A22}

D, H: *eyFLP, UAS-mCD8-GFP /+ ;; tub-GAL4, FRT82B, tub-GAL80/FRT82B, crb*^{11A22}

Fig 6

A,D,G,J: B, F: eyFLP, UAS-mCD8-GFP /+ ; UAS-RhoGEF2/ +; tub-GAL4, FRT82B, tub-GAL80/FRT82B

B,E,H,K: eyFLP, UAS-mCD8-GFP /+ ; UAS-RhoGEF2/ +; tub-GAL4, FRT82B, tub-GAL80/FRT82B, crb^{11A22}

Fig 7

A: *eyFLP, UAS-mCD8-GFP /+ ;; tub-GAL4, FRT82B, tub-GAL80/+*

B: eyFLP, UAS-mCD8-GFP /+ ;; tub-GAL4, FRT82B, tub-GAL80/FRT82B, crb^{11A22}

Supp Fig 1

A: *eyFLP, UAS-mCD8-GFP /+ ;; tub-GAL4, FRT82B, tub-GAL80/+*

B: *eyFLP, UAS-mCD8-GFP /+ ;; tub-GAL4, FRT82B, tub-GAL80/ FRT82B, UAS-Ptp10D-RNAi*^{1101}

C: *eyFLP, UAS-mCD8-GFP /+; UAS-Ptp10D-RNAi*^{1102} */ + ; tub-GAL4, FRT82B, tub-GAL80/ FRT82B*

D: *eyFLP, UAS-mCD8-GFP /+ ;; tub-GAL4, FRT82B, tub-GAL80/ FRT82B, scrib*^{1}*, UAS-Ptp10D-RNAi*^{1101}

E: *eyFLP, UAS-mCD8-GFP /+; UAS-Ptp10D-RNAi*^{1102} */ + ; tub-GAL4, FRT82B, tub-GAL80/ FRT82B, scrib*^{1}

F: *eyFLP, UAS-mCD8-GFP /+ ;; tub-GAL4, FRT82B, tub-GAL80/ FRT82B, scrib*^{1}

Supp Fig 2

A, D: eyFLP, UAS-mCD8-GFP /+ ;; tub-GAL4, FRT82B, tub-GAL80/+

B, E: y-w-, eyFLP2/+; Act>GAL4, UAS-GFP/+; tubGAL80, FRT82B, scrib1/FRT82B

C, F: y-w-, eyFLP2/+; Act>GAL4, UAS-GFP/+; tubGAL80, FRT82B, scrib1/FRT82B, UAS-sas-RNAi^{39086}

Supp Fig 3

A: *eyFLP, UAS-mCD8-GFP /+ ;; tub-GAL4, FRT82B, tub-GAL80/+*

B: *eyFLP, UAS-mCD8-GFP /+ ; UAS-RhoGEF2/+; tub-GAL4, FRT82B, tub-GAL80/UAS- Dicer2*

C: *eyFLP, UAS-mCD8-GFP /+ ; UAS-RhoGEF2/+; tub-GAL4, FRT82B, tub-GAL80/UAS-Ptp10D-RNAi*^{1101}

D: *eyFLP, UAS-mCD8-GFP /+ ;; tub-GAL4, FRT82B, tub-GAL80/UAS-Ptp10D-RNAi*^{1101}

Journal of Cell Science • Supplementary information

Supp Fig 4

A: *eyFLP, UAS-mCD8-GFP /+ ;; tub-GAL4, FRT82B, tub-GAL80/+*

B: *eyFLP, UAS-mCD8-GFP /+ ; UAS-RhoGEF2/+; tub-GAL4, FRT82B, tub-GAL80/UAS- Dicer2*

C: *eyFLP, UAS-mCD8-GFP /+ ; UAS-RhoGEF2/+; tub-GAL4, FRT82B, tub-GAL80/UAS-Ptp10D-RNAi*^{1101}

D: *eyFLP, UAS-mCD8-GFP /+ ;; tub-GAL4, FRT82B, tub-GAL80/UAS-Ptp10D-RNAi*^{1101}

Supp Fig 5

A, E: *eyFLP, UAS-mCD8-GFP /+ ;; tub-GAL4, FRT82B, tub-GAL80/+*

B, F: *eyFLP, UAS-mCD8-GFP /+ ; UAS-RhoGEF2/+; tub-GAL4, FRT82B, tub-GAL80/UAS- Dicer2*

C, G: *eyFLP, UAS-mCD8-GFP /+ ; UAS-RhoGEF2/+; tub-GAL4, FRT82B, tub-GAL80/UAS-Ptp10D-RNAi*^{1101}

D, H: *eyFLP, UAS-mCD8-GFP /+ ;; tub-GAL4, FRT82B, tub-GAL80/UAS-Ptp10D-RNAi*^{1101}

Supp Fig 6

A: *eyFLP, UAS-mCD8-GFP /+ ;; tub-GAL4, FRT82B, tub-GAL80/+*

B: *eyFLP, UAS-mCD8-GFP /+ ; UAS-RhoGEF2/+; tub-GAL4, FRT82B, tub-GAL80/UAS- Dicer2*

Supp Fig 7

A, E: *eyFLP, UAS-mCD8-GFP /+ ;; tub-GAL4, FRT82B, tub-GAL80/+*

B, F: *eyFLP, UAS-mCD8-GFP /+ ; UAS-RhoGEF2/+; tub-GAL4, FRT82B, tub-GAL80/UAS- Dicer2*

C, G: *eyFLP, UAS-mCD8-GFP /+ ; UAS-RhoGEF2/+; tub-GAL4, FRT82B, tub-GAL80/UAS-Ptp10D-RNAi*^{1101}

D, H: *eyFLP, UAS-mCD8-GFP /+ ;; tub-GAL4, FRT82B, tub-GAL80/UAS-Ptp10D-RNAi*^{1101}

Supp Fig 8

A,C,E,G: eyFLP, UAS-mCD8-GFP /+ ;; tub-GAL4, FRT82B, tub-GAL80/+

B,D,F,H: eyFLP, UAS-mCD8-GFP /+ ;; tub-GAL4, FRT82B, tub-GAL80/FRT82B, crb^{11A22}

Supp Fig 9

A, E: *eyFLP, UAS-mCD8-GFP /+ ;; tub-GAL4, FRT82B, tub-GAL80/+*

B, F: *eyFLP, UAS-mCD8-GFP /+ ; UAS-RhoGEF2/ +; tub-GAL4, FRT82B, tub-GAL80/FRT82B*

Journal of Cell Science • Supplementary information

Supp Fig 10

Panel 1: *eyFLP, UAS-mCD8-GFP /+ ;; tub-GAL4, FRT82B, tub-GAL80/+*

Panel 2: *eyFLP, UAS-mCD8-GFP /+ ;; tub-GAL4, FRT82B, tub-GAL80/ FRT82B, scrib^{1}*

Panel 3: *eyFLP, UAS-mCD8-GFP /+; UAS-Ptp10D UAS.cTa /+ ; tub-GAL4, FRT82B, tub-GAL80/FRT82B, scrib^{1}*

Panel 4: *eyFLP, UAS-mCD8-GFP /+ ;; tub-GAL4, FRT82B, tub-GAL80/ FRT82B, scrib^{1}, UAS-Ptp10D-RNAi^{1101}*

Table S2. *Drosophila* food recipes

Reagents	Molasses based food recipe (g/L)	/Low protein diet (g/L)
Molasses*	93	-
Yeast	60	10.7
Agar	5	4.8
Glucose (Dextrose)	10.6	47.6
Sugar (Raw)	-	23.8
Semolina	88	59.5
Potassium Sodium Tartrate Tetrahydrate	-	7.14
Calcium Chloride Dihydrate	-	0.44
	ml/L	ml/L
Tesgosept solution (10 w/v% methyl 4-hydroxybenzoate, 0.5 v/v% 100% EtOH)	17.46	7.14
Propionic acid mix	9.2	3.57

Journal of Cell Science • Supplementary information

(41.2 v/v% 99% propionic acid, 4.2 v/v%
85% phosphoric acid)

*We used Bundaberg food-grade molasses, which like most molasses products, typically contains 40-55% sugar. This sugar content is mainly composed of Sucrose, Glucose and Fructose.

Journal of Cell Science • Supplementary information
